# Protein phosphatase SCP4 regulates cartilage development and endochondral osteogenesis via FoxO3a dephosphorylation

**DOI:** 10.1111/cpr.13691

**Published:** 2024-06-17

**Authors:** Pinger Wang, Kaiao Zou, Jin Cao, Zhengmao Zhang, Wenhua Yuan, Jiali Chen, Jianbo Xu, Zhen Zou, Di Chen, Hongfeng Ruan, Jianying Feng, Xia Lin, Hongting Jin

**Affiliations:** ^1^ Institute of Orthopedics and Traumatology The First Affiliated Hospital of Zhejiang Chinese Medical University, Zhejiang Provincial Hospital of Chinese Medicine Hangzhou Zhejiang China; ^2^ The First College of Clinical Medicine Zhejiang Chinese Medical University Hangzhou Zhejiang China; ^3^ The MOE Key Laboratory of Biosystems Homeostasis and Protection and Zhejiang Provincial Key Laboratory of Cancer Molecular Cell Biology, Life Sciences Institute Zhejiang University Hangzhou Zhejiang China; ^4^ Department of Pathology and Laboratory Medicine Weill Cornell Medicine New York City New York USA; ^5^ Research Center for Computer‐aided Drug Discovery Chinese Academy of Sciences, Shenzhen Institute of Advanced Technology Shenzhen Guangdong China; ^6^ Faculty of Pharmaceutical Sciences Chinese Academy of Sciences, Shenzhen Institute of Advanced Technology Shenzhen Guangdong China; ^7^ School of Stomatology Zhejiang Chinese Medical University Hangzhou Zhejiang China; ^8^ Department of Hepatobiliary and Pancreatic Surgery and Zhejiang Provincial Key Laboratory of Pancreatic Disease, The First Affiliated Hospital Zhejiang University School of Medicine Hangzhou Zhejiang China

## Abstract

The regulatory mechanisms involved in embryonic development are complex and yet remain unclear. SCP4 represents a novel nucleus‐resident phosphatase identified in our previous study. The primary aim of this study was to elucidate the function of SCP4 in the progress of cartilage development and endochondral osteogenesis. *SCP4* ^*−/−*^ and *SCP4* ^*Col2ER*^ mice were constructed to assess differences in bone formation using whole skeleton staining. ABH/OG staining was used to compare chondrocyte differentiation and cartilage development. Relevant biological functions were analysed using RNA‐sequencing and GO enrichment, further validated by immunohistochemical staining, Co‐IP and Western Blot. Global *SCP4* knockout led to abnormal embryonic development in *SCP4* ^*−*/*−*^ mice, along with delayed endochondral osteogenesis. In parallel, chondrocyte‐specific removal of *SCP4* yielded more severe embryonic deformities in *SCP4* ^*Col2ER*^ mice, including limb shortening, reduced chondrocyte number in the growth plate, disorganisation and cell enlargement. Moreover, RNA‐sequencing analysis showed an association between SCP4 and chondrocyte apoptosis. Notably, Tunnel‐positive cells were indeed increased in the growth plates of *SCP4* ^*Col2ER*^ mice. The deficiency of SCP4 up‐regulated the expression levels of pro‐apoptotic proteins both in vivo and in vitro. Additionally, phosphorylation of FoxO3a (pFoxO3a), a substrate of SCP4, was heightened in chondrocytes of *SCP4* ^*Col2ER*^ mice growth plate, and the direct interaction between SCP4 and pFoxO3a was further validated in chondrocytes. Our findings underscore the critical role of SCP4 in regulating cartilage development and endochondral osteogenesis during embryonic development partially via inhibition of chondrocytes apoptosis regulated by FoxO3a dephosphorylation.

## Graphical abstract

A noval protein phosphatase SCP4 regulates cartilage development and endochondral osteogenesis through the apoptotic pathway during embryonic development. SCP4 could direct interaction with pFoxO3a and dephosphorylated it. The deficiency of SCP4 resulted in the inactivation of FoxO3a, leading to growth plate chondrocytes apoptosis and abnormal embryonic development.

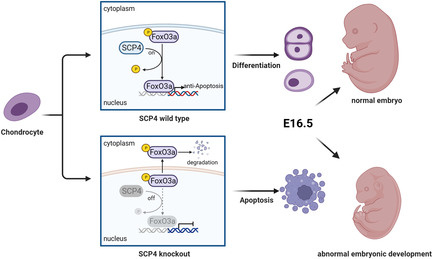

## 1 INTRODUCTION

Endochondral osteogenesis represents a pivotal mode of bone development and is instrumental in the formation of the majority of long bones in the extremities and spine.^1^, ^2^ During embryonic limb development, mesenchymal progenitor cells are regulated by a series of factors to transition into mature chondrocytes.^3^ These maturing chondrocytes become organised into distinct zones within the epiphyseal growth plate—namely, a resting zone (RZ), a proliferating zone (PZ) and a hypertrophic zone (HZ).^4^ Ossification occurs as terminally differentiated chondrocytes perish, leading to calcification. This calcified cartilage is invaded by blood vessels, along with osteoclasts and osteoblast progenitors, initiating the formation of the primary ossification centre.^5^ More precisely, the limb lengthening depends on the proliferation and hypertrophy of chondrocytes and the ossification process of the endosteal layer determines whether the skeleton develops normally.^6^, ^7^ The occurrence of any abnormalities, such as diminished chondrocyte proliferation, heightened chondrocyte apoptosis, abnormal chondrocyte differentiation and delayed onset of mineralisation, eventually lead to deformities or delays in embryonic bone development.^8^, ^9^

An array of phosphatases involved in the orchestration of cartilage development and endochondral osteogenesis.^10^, ^11^, ^12^ For instance, the activation of protein phosphatase 2A (PP2A) by the parathyroid hormone‐related peptide (PTHrP)/cAMP/protein kinase A (PKA) cascade in chondrocytes dephosphorylates of histone deacetylase 4 (HDAC4), thereby amplifying its activity and enhances its nuclear localization to regulate chondrocyte hypertrophy and endochondral bone formation.^13^, ^14^ The lipid phosphatase SH2‐containing inositol‐5′‐phosphatase‐1 (SHIP‐1) in osteochondroprogenitor cells can positively regulate chondrocyte hypertrophy and skeletal development by initiating phosphatidylinositol‐3′‐kinase (PI3K) signalling.^12^, ^15^ In contrast, the deletion of protein phosphatase 5 (PP5) promotes bone and cartilage development along with increased levels of phospho‐PPARγ.^10^ Here, we discovered a novel nuclear protein phosphatase, small C‐terminal domain phosphatase‐4 (SCP4), elucidating its pivotal role in orchestrating cartilage development and endochondral osteogenesis.

SCP4, also known as carboxy‐terminal domain (CTD) small phosphatase like 2 (CTDSPL2), act as a metal‐dependent serine/threonine phosphatase capable of directly dephosphorylating various targets and interacting with multiple nuclear proteins.^16^ Notably, SCP4 operates as a chromatin‐associated CTD phosphatase in humans and is hypothesized to participate in gene regulation during erythroid differentiation.^17^ The indispensability of SCP4 for growth and development is emphasised by the survival of *SCP4* knockout (*SCP4* ^*−/−*^) mice during the perinatal period through maternal glycogen supply, yet their subsequent demise within 24 h of birth due to an inability to produce glycogen.^18^ In previous studies, we have reported in previous studies that SCP4 can turn off Smad‐mediated transcriptional activation in the BMP signalling pathway through the dephosphorylation of Smad1/5/8, a process essential for skeletogenesis.^14^, ^19^ In addition, SCP4 can dephosphorylate Snail to suppress the ubiquitin‐dependent proteasomal degradation of Snail, leading to an augmentation of TGFβ‐induced epithelial interstitial transformation.^20^ Furthermore, SCP4's ability to dephosphorylate Forkhead Box O1 (FoxO1) and Forkhead Box O3a (FoxO3a) within the nucleus promotes the nuclear retention of FoxO1 and FoxO3a, culminating in the induction of Glucose‐6‐phosphate dehydrogenase and other enzymes for gluconeogenesis.^18^ Although the above clues have implied the possible involvement of SCP4 in regulating bone formation and metabolism, its specific role in regulating endochondral osteogenesis and cartilage development remains to be fully characterised.

In this study, we demonstrated that in vivo knockout of the SCP4 gene severely affected cartilage development, endochondral ossification and skeletal development, especially evident in chondrocyte conditional *SCP4* knockout mice. Through the utilisation of RNA‐sequencing bioinformatics analysis and experimental validation, we mechanistically established that *SCP4* knockout increased the phosphorylation of FoxO3a, consequently promoting chondrocyte apoptosis. Hence, this study revealed a novel function of SCP4 in regulating endochondral ossification and bone formation.

## 2 MATERIALS AND METHODS

### 2.1 Animals

*SCP4* ^*−/−*^ mice were generated as previously described.^18^ *SCP4* ^*f/f*^ mice (Shanghai Biomodel Organism Science and Technology Development Co., Ltd) on a C57BL/6J mice background were crossed with Col2‐CreERT2 mice to generate chondrocyte conditional *SCP4* knockout (*SCP4* ^*Col2ER*^) mice. Col2‐CreER^T2^ mice were gifts from Dr. Di Chen's laboratory (Research Center for Human Tissues and Organs Degeneration, Shenzhen Institutes of Advanced Technology, Chinese Academy of Sciences, Shenzhen, China). To activate CreER^T2^, a single intraperitoneal injection of tamoxifen (Sigma–Aldrich, T5648) (100 mg/kg in corn oil), was administered to female mice at 12.5 days of pregnancy. All experimental protocols for mice were approved by the Animal Experimentation Ethics Committee of Zhejiang Chinese Medical University and complied with the guidelines of the Care and Use of Laboratory Animals (20221121–06).

### 2.2 Whole‐mount skeleton staining

To visualise the cartilage and bone of the entire skeleton, 16.5 day and 18.5 day mouse embryos (E16.5 and E18.5) were sequentially stained with Alcian blue (for cartilage) and Alizarin red (for bone) using the standard protocol. Briefly, after fixation with 95% ethanol for 24 h, embryos were stained with 0.03% (w/v) Alcian blue in 80% ethanol and 20% acetic acid solution for 1 day, followed by 0.03% (w/v) Alizarin red in 1% KOH solution for another 24 h. The embryos were then kept in 1% KOH and glycerol (gradient ratio: 50%, 80%, 100%) until analysis.

### 2.3 Histological staining

For histological studies, all embryo samples were fixed in 4% paraformaldehyde for 1 day and decalcified in 14% ethylene diamine tetraacetic acid (EDTA) solution (pH = 7.2) for 3 days. Subsequently, the specimens were dehydrated and embedded in paraffin wax. Paraffin blocks were cut into 4‐μm‐thick slices for staining. After deparaffinisation and rehydration, the sections were stained with Alcian Blue Hematoxylin/Orange G (ABH/OG) for morphological observation.

### 2.4 TUNEL assay

To evaluate the apoptotic cells in the cartilage, TUNEL assay was conducted using a TUNEL staining kit (Beyotime, C1088) according to the manufacturer's protocol. Briefly, following deparaffinisation and rehydration, sections were permeabilized with DNase‐free Proteinase K (20 μg/mL) for 30 min at 37°C. Then, the samples were treated with TUNEL and incubated at 37°C for 1 h in the dark. The slides were washed with PBS three times, and the cell nuclei were stained with 4′,6‐diamidino‐2‐phenylindole. All sections were observed under a laser scanning microscope.

### 2.5 Immunohistochemical staining

Standard IHC procedure was used. Briefly, tissue sections were deparaffinised, followed by antigen retrieval in sodium citrate solution and then incubated with 0.3% Triton X‐100 for 10 min at room temperature. After that, the samples were treated with an endogenous peroxidase blocker (ZSGB‐BIO, PV‐9001) at room temperature for 10 min and probed with primary antibodies at 4°C overnight. After extensive washing, the tissue sections were incubated with corresponding secondary antibodies for 20 min. Positive staining was developed with diaminobenzidine solution (ZSGB‐BIO, PV‐9001), while CAT haematoxylin was used for counterstaining. The primary antibodies used in this study included Caspase‐3 (ABclonal, A2156), active‐Caspase‐3 (HuaBio, ET1608‐64) Caspase‐8 (ABclonal, A0215), Caspase‐9 (HuaBio, ET1603‐27), pFoxO3a (Cell Signalling Technology, CST‐13129s), FoxO3a (ABclonal, A0102) and pFoxO1 (Cell Signalling Technology, CST‐84192s).

### 2.6 Cell culture

Primary articular chondrocytes were isolated from the cartilage of the neonatal anterior rib cage of *SCP4* ^*f/f*^ mice. Specifically, *SCP4* ^*f/f*^ mice were sacrificed and disinfected in 75% ethyl alcohol before sampling. Then, the anterior rib cage was digested by 3 mg/mL Collagenase‐D (Roche) in DMEM Nutrient Mixture F‐12 (DME/F‐12) medium (Thermo Fisher Scientific) containing 1% penicillin/streptomycin (Thermo Fisher Scientific) for 6 h at 37°C with intermittent percussion. Following digestion, the chondrocytes were harvested and cultured in a complete DME/F‐12 medium supplemented with 10% FBS. ATDC5 cells (RIKEN Cell Bank, Tsukuba, Japan) were cultured in a DME/F12 medium containing 10% FBS.

### 2.7 Lentivirus/adenovirus transfection

The adenovirus Ad‐Con, Ad‐GFP, Ad‐Cre and Ad‐SCP4 as well as the lentivirus Lv‐Con, Lv‐FoxO1, Lv‐FoxO3a and Lv‐SCP4D294N (GeneChem, Shanghai, China) were transfected when 80% density of adherent cells. The viruses with MOI = 100 or 50 were added to a DME/F12 medium containing 2% FBS and replaced the medium to a DME/F12 medium containing 10% FBS at 12 h of transfection. After 72 h of transfection, discarded supernatants and collected cells (adenovirus MOI = 100, lentivirus MOI = 50). The transfection efficiency was assessed by Western blotting of the target proteins.

### 2.8 RNA‐sequencing analysis

RNA‐seq analysis of Ad‐Cre or Ad‐GFP transfected *SCP4* ^*f/f*^ mice primary chondrocytes was conducted. Briefly, total RNA was extracted using a RNeasy Micro Kit (QIAGEN) with TRIzol reagent (Invitrogen, Carlsbad, CA, USA). The concentration and quality of RNAs were assessed by an Agilent 2100 Bioanalyzer (Agilent Technologies) and a Nanodrop 2000 (Thermo, Waltham, MA, USA). mRNA‐seq libraries were prepared using the VAHTS TM mRNA‐seq V2 Library Prep Kit for Illumina (Vazyme, #NR601) and then run on an Illumina HiSeq X‐Ten next‐generation sequencer. FastQC (v 0.11.9) was used for quality control and trimmed reads (http://www.bioinformatics.bbsrc.ac.uk/projects/fastqc). Transcripts expressed at or above 1 count per million reads in our samples were selected for analysis. Differentially expressed genes (DEGs) between control and SCP4‐depleted cells were identified by a linear regression model package. GO analyses were performed using DAVID^21^ and WebGestalt.^22^

### 2.9 Immunocoprecipitation (Co‐IP) assay

Endogenous Co‐IP was carried out to analyse the interaction between SCP4 and FoxO3a in primary rib chondrocytes from *SCP4* ^*f/f*^ mice. Cells were lysed with cell lysis buffer (Beyotime, catalogue P0013) at 4°C for 30 min, and the cell lysates were cleared by centrifugation at 12,000 rpm for 15 min. The supernatant was incubated with control IgG Ab (ABclonal, AC005) or anti‐SCP4 (Novus, NBP1‐91814) overnight at 4°C, the immune complex was precipitated with protein A+G Agarose beads (Beyotime, catalogue P2055), and at 4°C for another 4 h. The Co‐IP of SCP4 and FoxO3a was examined by SDS–PAGE and Western blotting.

### 2.10 Western blot analysis

Standard Western blot protocol was employed. Briefly, cells were lysed with RIPA lysis buffer supplemented with phosphatase and protease inhibitors (Roche Diagnostics, Basel, Switzerland). Total proteins were quantified and separated by 10 or 15% SDS‐PAGE and transferred onto 0.22 μm NC membranes (Millipore, Burlington, MA, USA). The membranes were then blocked with 5% skim milk (Beyotime, P0216) at room temperature (RT = 25°C) for 1 h and incubated with primary antibodies against active‐Caspase‐3 (HuaBio, ET1608‐64), Caspase‐3 (ABclonal, A2156), Caspase‐8 (ABclonal, A0215), Caspase‐9 (HuaBio, ET1603‐27), CTDSPL2 (Novus, NBP1‐91814), pFoxO3a (Cell Signalling Technology, CST‐13129s), FoxO3a (ABclonal, A0102), pFoxO1 (Cell Signalling Technology, CST‐84192s), FoxO1 (Cell Signalling Technology, CST‐2880s) and β‐actin (Proteintech, HRP‐60008). The membranes were washed with Tris‐buffered saline (TBS)‐0.1% Tween 20 (TBST) and subsequently incubated with anti‐rabbit or anti‐mouse IgG (H + L) secondary antibody (IRDye®800CW Goat anti‐Rabbit 962–32,211, LI‐COR Biosciences, 1:20,000; IRDye®680RD Goat anti‐Mouse 926–68,070, LI‐COR Biosciences, 1:20,000) for 1 h at RT in the dark. After washing in TBST, protein immunoreactivity was detected using the Odyssey Fluorescence Imaging system (LI‐COR Biosciences, Lincoln, NE, USA). Western blot with β‐actin was used as a loading control.

### 2.11 Statistical analysis

All values were expressed as the mean ± standard analysis (including unpaired deviations). Statistical tests were performed using GraphPad Prism 8.2 software. Student's *t*‐test was used to compare the two groups. One‐way analysis of variance (ANOVA) was applied for statistical analysis for comparison of more than two groups. *p* < 0.05 was considered statistically significant.

## 3 RESULTS

### 3.1 Deletion of *SCP4* induces abnormal skeletal development in mice

**FIGURE 1:**
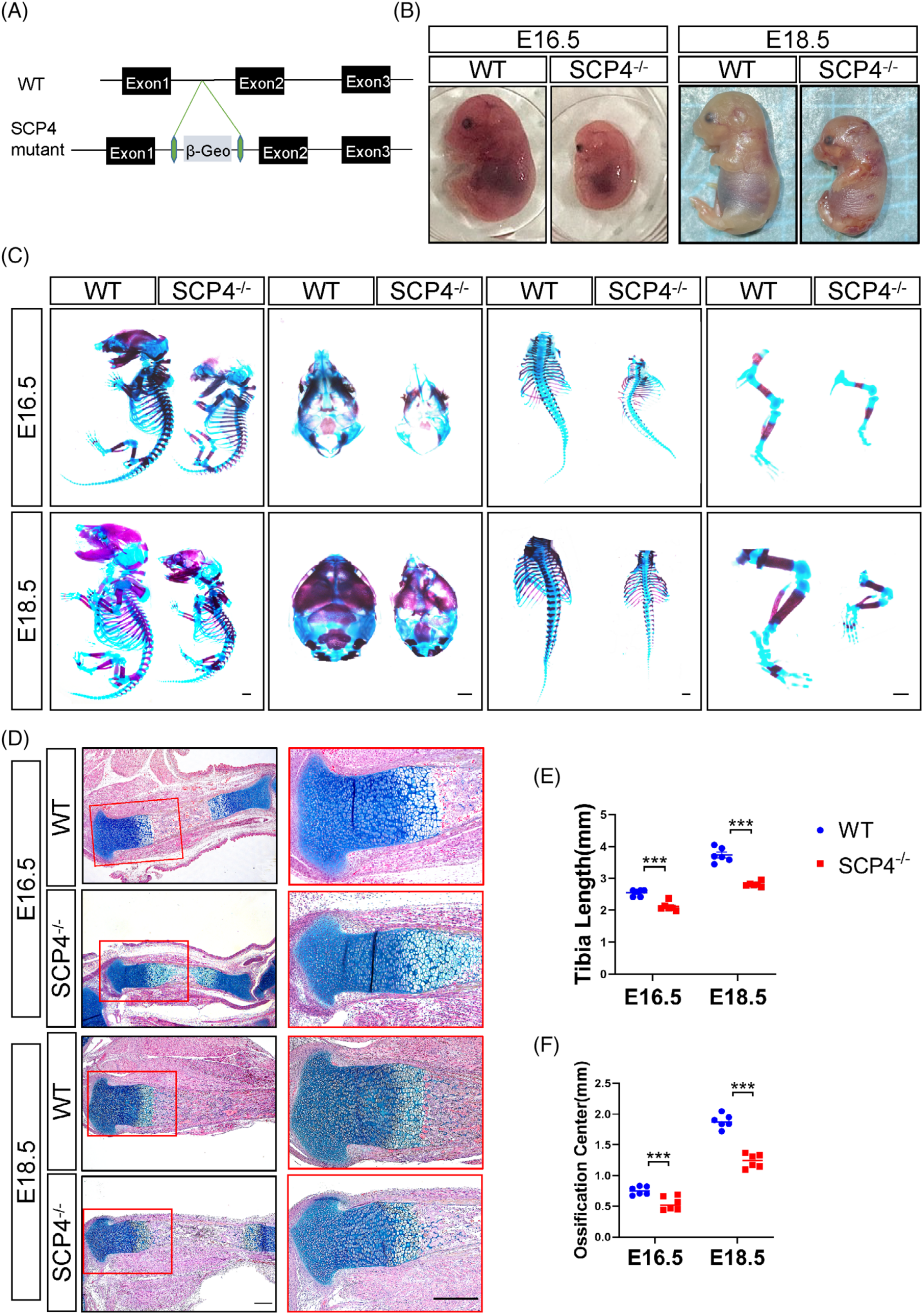
Delayed skeletal development in *SCP4* knockout mice. (A) Representative results of PCR screening for the *SCP4* null allele. WT: wild type. (B) Gross appearance of WT and *SCP4* ^*−/−*^ mice at E16.5 and E18.5. (C) Whole‐mount skeletal staining of WT and *SCP4* ^*−/−*^ mouse using Alizarin red and Alcian blue. Scale bar: 1 mm. (D) ABH/OG staining of E16.5 and E18.5 tibial. Scale bar: 0.5 mm. (E) Statistical analysis of limb length (*n* = 6). Statistical significance was determined by Student's *t‐*test and the results are shown as the mean ± SEM. *: *p* < 0.05, **: *p* < 0.005, ***: *p* < 0.001. *n* ≥ 3 in each group. (F) Quantification of the length of primary ossification centres in the tibia of *SCP4* ^*−/−*^ and WT mice (*n* = 6).

To investigate the physiological role of SCP4 in development, we constructed *SCP4* ^*−/−*^ mice (Figure 1A) and found that *SCP4* ^*−/−*^ mice exhibited a much smaller body size than their littermates at both E16.5 and E18.5 (Figure 1B). Consistently, whole‐mount skeleton staining of embryos at E16.5 and E18.5 revealed that the size of the skull thorax and spine in *SCP4* ^*−/−*^ mouse embryos, particularly the limbs, was significantly smaller than that of littermate controls (Figure 1C,E). Furthermore, ABH/OG staining also showed that markedly shorter physiological tibia length in *SCP4* ^*−/−*^ mice delayed primary ossification centre formation at E16.5 and E18.5 mice (Figure 1D,F). These results suggest that deletion of SCP4 may lead to abnormal skeletal development.

### 3.2 Chondrocyte‐specific knockout of *SCP4* leads to delayed limb development in the embryo

**FIGURE 2:**
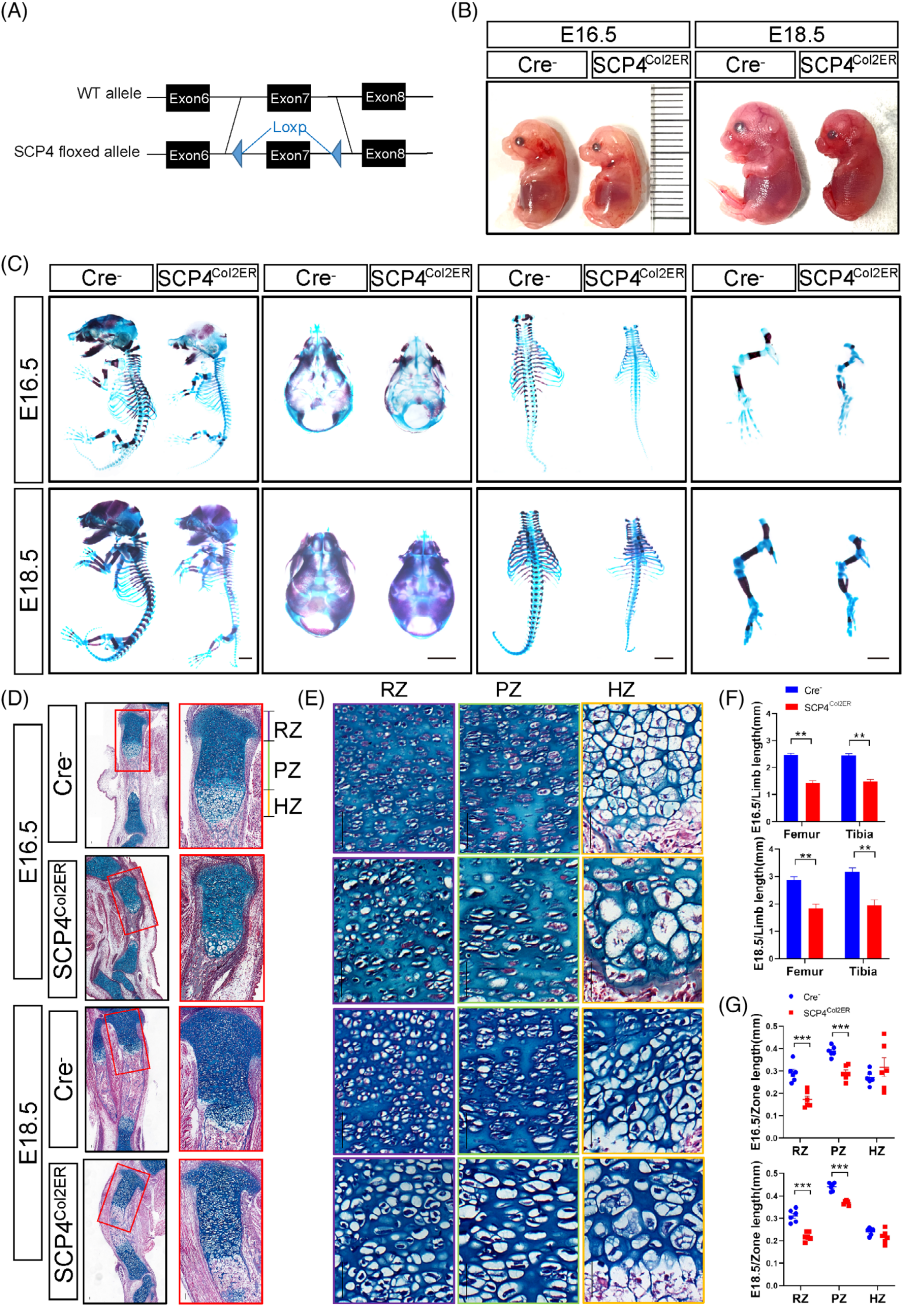
Cartilage development and endochondral osteogenesis abnormalities in chondrogenic precursor cell‐specific knockout *SCP4* mice. (A) Location of two Loxp loci in the *SCP4* allele. (B) Gross appearance of Cre^*−*^ and *SCP4* ^*Col2ER*^ mice at E16.5 and E18.5. (C) Whole‐mount skeletal staining of Cre^*−*^ and *SCP4* ^*Col2ER*^ mouse using Alizarin red and Alcian blue. Scale bar: 1 mm. (D,E) ABH/OG staining of E16.5 and E18.5 tibial. Scale bar: 0.05 mm. (F) Statistical analysis of limb length. (G) Statistical analysis of the length of RZ, PZ and HZ. Statistical significance was determined by Student's *t‐*test, and the results are shown as the mean ± SEM. *: *p* < 0.05, **: *p* < 0.005, ***: *p* < 0.001. *n* ≥ 5 in each group.

To specifically determine SCP4's role in endochondral skeletal development in vivo, we generated chondrocyte‐specific *SCP4* knockout mice, *SCP4* ^*Col2ER*^ (Figure 2A) and dissected embryos at 16.5 and 18.5 days. Comparable to *SCP4* ^*−/−*^ mice (Figure 2B), *SCP4* ^*Col2ER*^ mice showed delayed developmental and reduced ossification levels in the head, spine and lower limbs (Figure 2C). In particular, *SCP4* ^*Col2ER*^ mice displayed delayed endochondral bone formation in the tibial tissue sections compared to their Cre‐negative control littermates (Figure 2D,F). The extension of primary ossification centre lengthening in *SCP4* ^*Col2ER*^ mice was also clearly delayed (Figure S1A). Chondrocytes in the RZ, PZ and HZ were hypocellular and disorganised in *SCP4* ^*Col2ER*^ mice compared with their littermates (Figure 2E). Interestingly, the hypertrophic chondrocytes and, to a lesser degree, the resting and proliferative chondrocytes in *SCP4* ^*Col2ER*^ mice appeared enlarged with an expanded cytoplasm (Figure 2E). The growth plate cartilage development is also noticeably affected, with shortened resting and proliferation regions in *SCP4* ^*Col2ER*^ mice (Figures S1B and 2G). These findings underscore SCP4's pivotal role in chondrocyte development and endochondral ossification.

### 3.3 Loss of SCP4 in chondrocytes enhances chondrocyte apoptosis

**FIGURE 3:**
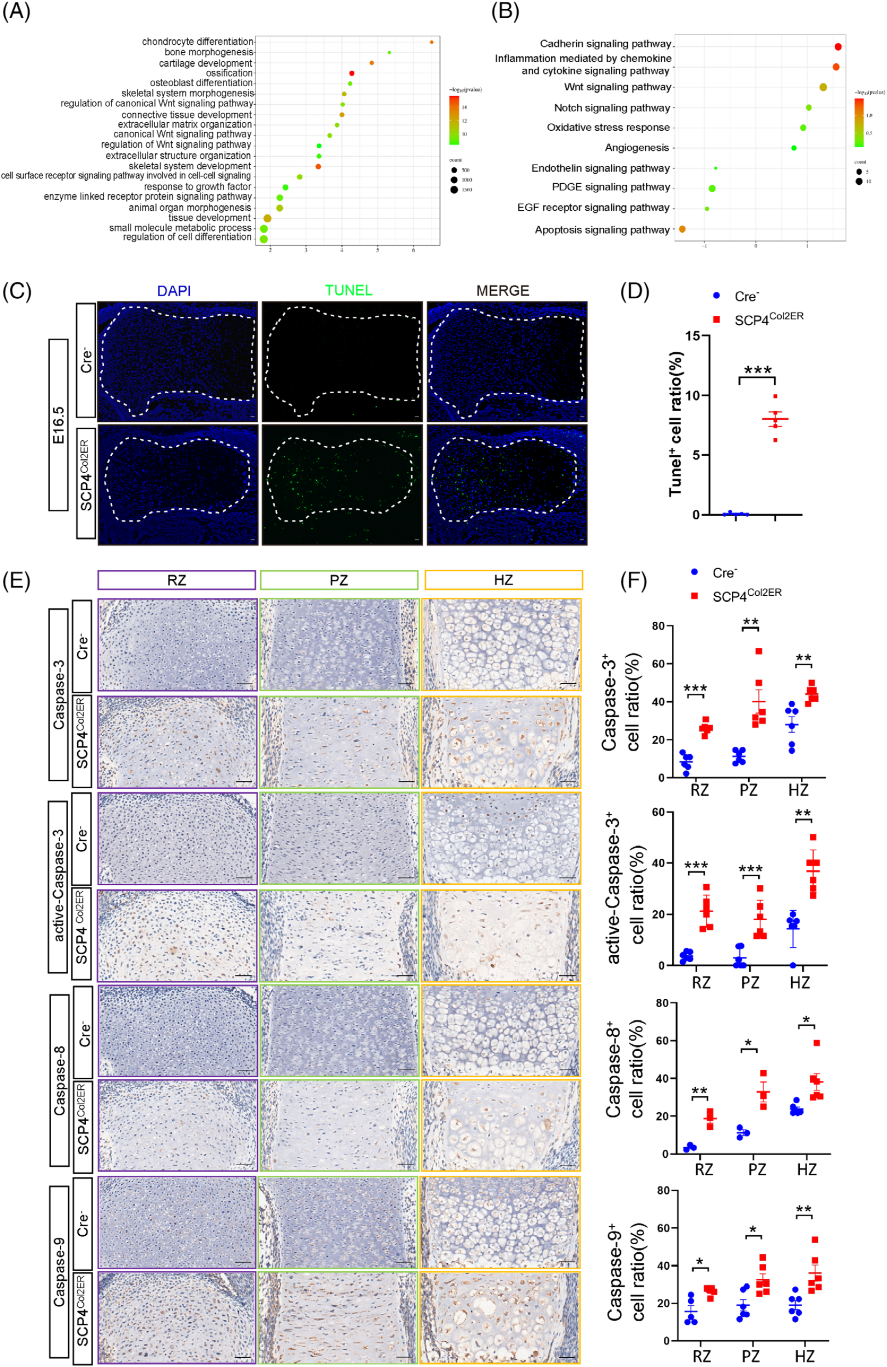
Significantly enhanced chondrocyte apoptosis in *SCP4* ^*Col2ER*^ mice. (A) Top 20 GO terms of biological pathways. (B) Panther pathway enrichment top 10. (C) TUNEL staining (green). Scale bar: 0.1 mm. (D) Quantification of TUNEL‐positive cells. (E) Immunohistochemical staining of apoptosis markers on sections of Cre^*−*^ and *SCP4* ^*Col2ER*^ mouse tibial growth plates at E16.5. Scale bar: 0.05 mm. (F) Quantification of Caspase‐3, active‐Caspase‐3, Caspase‐8 and Caspase‐9 positive cells. Statistical significance was determined by Student's *t*‐test, and the results are shown as the mean ± SEM. *: *p* < 0.05, **: *p* < 0.005, ***: *p* < 0.001. *n* ≥ 5 in each group.

To explore SCP4‐regulated pathways during skeletal development, we isolated primary chondrocytes from 3‐day‐old *SCP4* ^*f/f*^ mice and transfected these cells with either Cre recombinase adenovirus (Ad‐Cre) to induce *SCP4* depletion or GFP control adenovirus (Ad‐GFP) as a control, thus enabling in vitro analysis. Subsequently, RNA sequencing analysis was performed to screen DEGs resulting from SCP4 depletion (Figure S2A–C). And the top 20 GO terms enriched by differential expression of gene analysis involves the biological processes of chondrocyte differentiation, cartilage development, ossification and skeletal system development (Figure 3A). In addition, panther pathway enrichment analysis showed that *SCP4* silencing were mostly related to the apoptosis signalling pathway (Figure 3B). To confirm this result, we further performed TUNEL staining on the growth plate of E16.5 embryos to examine the apoptotic level of chondrocytes. Strikingly, we found an increased level of TUNEL‐positive cells throughout the cartilaginous area in *SCP4* ^*Col2ER*^ mice (Figure 3C,D). Moreover, the expression of important regulatory proteins involved in the apoptosis pathway, such as Caspase‐3, active‐Caspase‐3, Caspase‐8 and Caspase‐9, was also upregulated in the RZ/PZ/HZ areas of the epiphyseal growth plate of *SCP4* ^*Col2ER*^ mice (Figure 3E,F), suggesting that SCP4 loss in chondrocytes may trigger apoptotic signals, contributing to malformed embryonic skeletal development.

### 3.4 SCP4 regulates endochondral osteogenesis by dephosphorylating FoxO3a to inhibit chondrocytes apoptosis

**FIGURE 4:**
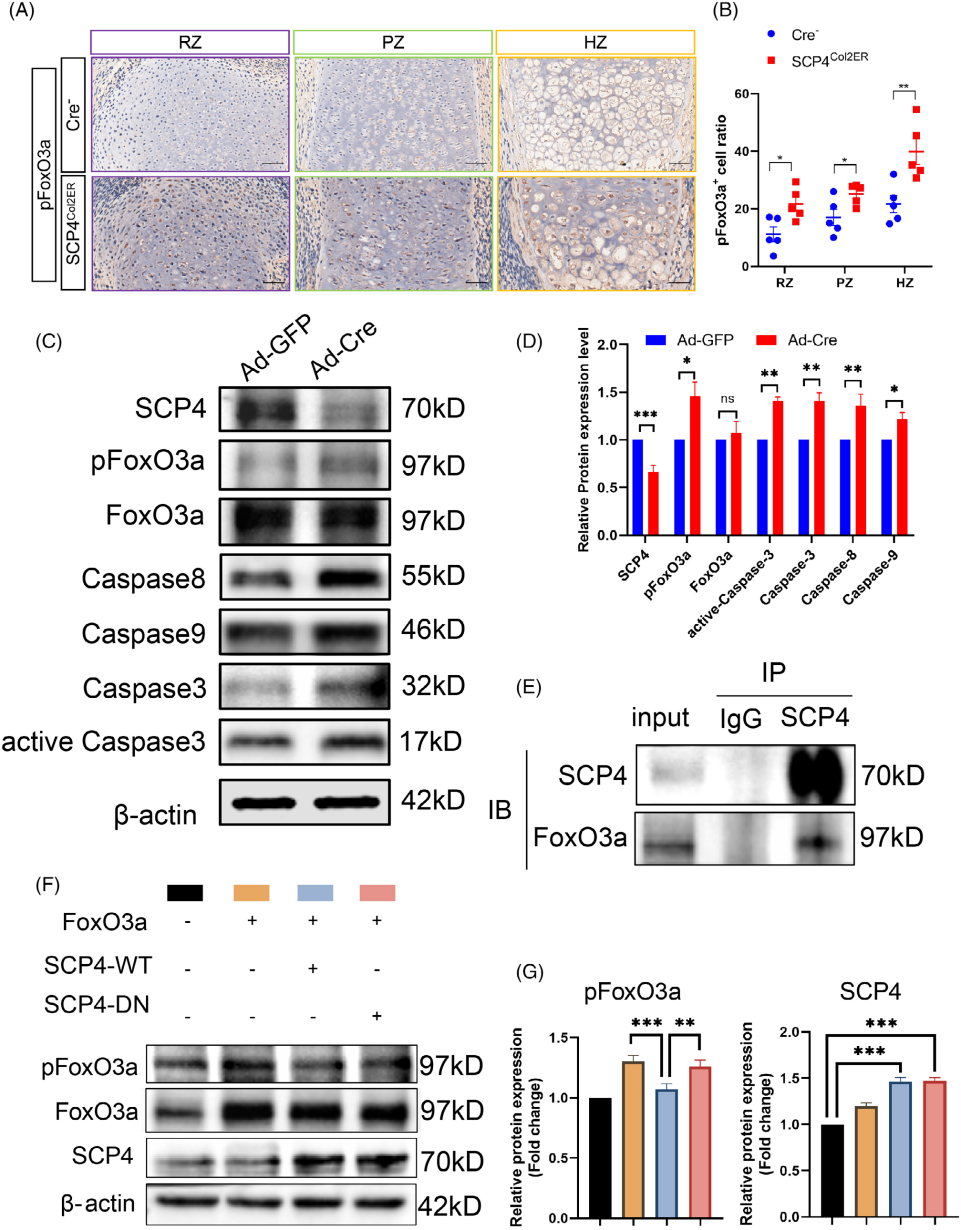
SCP4 dephosphorylates FoxO3a to inhibit chondrocytes apoptosis. (A) Immunohistochemical staining of pFoxO3a in tibial growth plate sections from Cre^*−*^ and *SCP4* ^*Col2ER*^ mice at E16.5. Scale bar: 0.05 mm. (B) Quantification of the pFoxO3a positive cells. (C) Western blot analysis of the indicated protein in the lysates of *SCP4* ^*f/f*^ primary chondrocytes transfected with Ad‐GFP or Ad‐Cre. Antibodies used were anti‐SCP4, pFoxO3a, FoxO3a, Caspase3, active‐Caspase‐3, Caspase8 and Caspase9. β‐actin was used as a loading control. (D) Quantification of the data from Western blot. (E) Co‐IP assay for the interactions between SCP4 and FoxO3a in primary rib chondrocytes. (F) SCP4 dephosphorylates pFoxO3a. ATDC5 cells were co‐transfected with Lv‐FoxO3a and Ad‐SCP4 (SCP4‐WT) or Lv‐SCP4‐DN. The levels of pFoxO3a, total FoxO3a and SCP4 were determined by Western blot. (G) Quantification of the data from Western blot. Statistical significance was determined by *t*‐test, and the results are shown as the mean ± SEM. *: *p* < 0.05, **: *p* < 0.005, ***: *p* < 0.001. *n* ≥ 3 in each group.

Our previous studies have identified several substrates of SCP4, including Smad1/5/8, Snail, FoxO1 and FoxO3a.^14^, ^18^, ^23^ Notably, FoxO1 and FoxO3a both members of the FoxO transcription factor family, play pivotal roles in multiple physiological processes, including the regulate of cell growth, differentiation and apoptosis.^24^, ^25^, ^26^, ^27^ Hence, we examined the phosphorylation levels of FoxO3a (pFoxO3a) and FoxO1 (pFoxO1) in *SCP4* ^*Col2ER*^ mice using immunohistochemical staining analysis and found that an enhanced pFoxO3a level in the RZ/PZ/HZ of the epiphyseal growth plate in *SCP4* ^*Col2ER*^ mice, while limited pFoxO3a expression in Cre‐negative mice (Figure 4A,B), indicating the crucial role of SCP4 in dephosphorylating FoxO3a in chondrocytes. Surprisingly, there was no significant difference in the expression of pFoxO1 in the epiphyseal growth plates between *SCP4* ^*Col2ER*^ mice and Cre‐negative mice (Figure S2D,E). Furthermore, we evaluated the impact of SCP4 on FoxO3a dephosphorylation in vitro and replicated the upregulated pFoxO3a levels in *SCP4*‐knockout chondrocytes (Figure 4C,D). Consistently, the expression of Caspase‐3, active‐Caspase‐3, Caspase‐8 and Caspase‐9 was elevated in vitro (Figure 4C,D). Notably, during skeletal development, we identified an interaction between SCP4 and FoxO3a using Co‐IP assay, suggesting the phosphatase activity of SCP4 towards FoxO3a in chondrocytes (Figure 4E).

**FIGURE 5:**
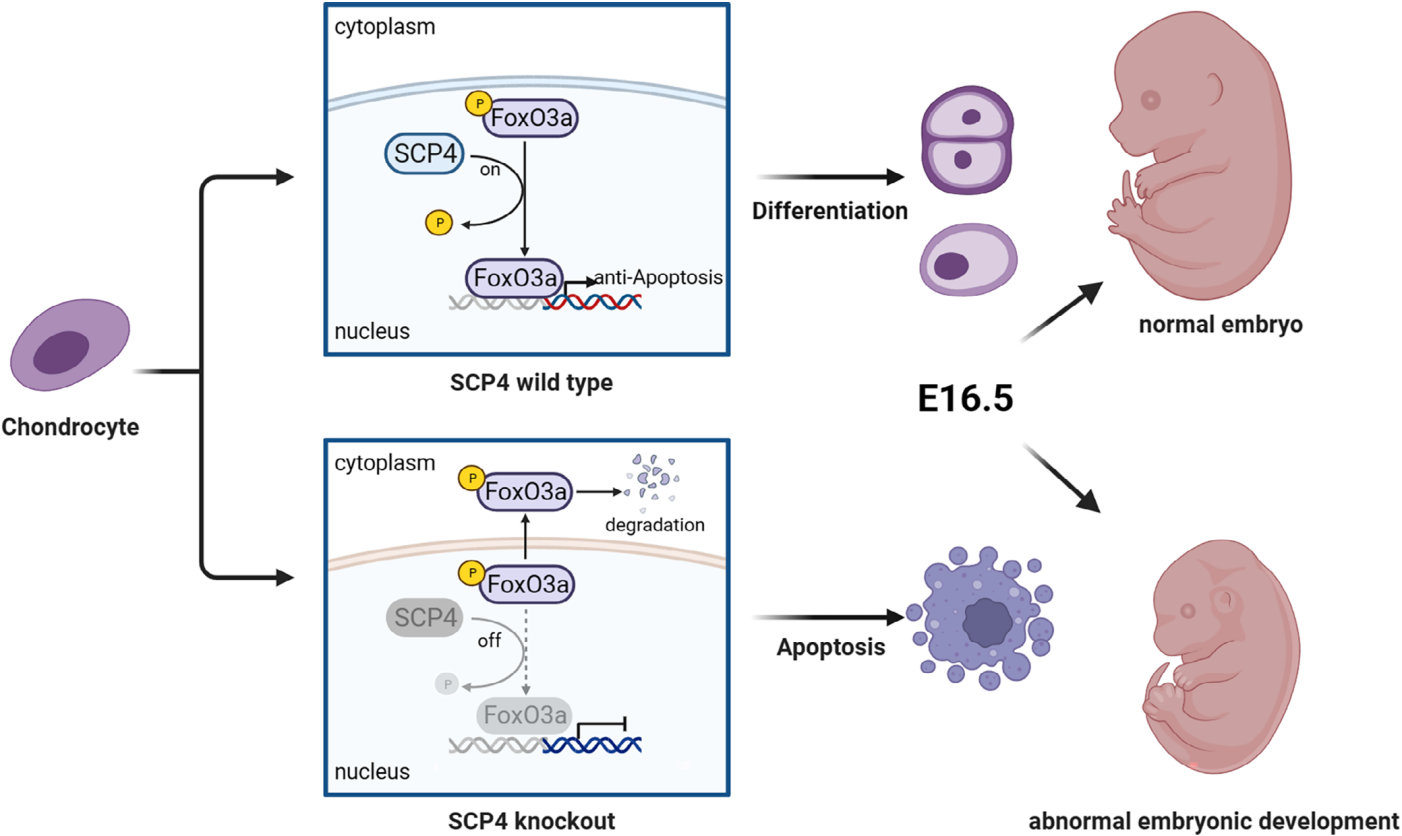
SCP4 could direct interaction with pFoxO3a and dephosphorylated it. The deficiency of SCP4 resulted in the inactivation of FoxO3a, leading to growth plate chondrocytes apoptosis and abnormal embryonic development.

To directly prove the necessity of SCP4's phosphatase activity in chondrocytes, we generated a phosphatase‐dead mutant of SCP4, in which the amino acid Asp 294 (D294) in the catalytic domain of SCP4 was mutated into Asn 294 (N294) (SCP4‐DN), consequently abolishing SCP4 phosphatase activity. Then, we co‐transfected wildtype SCP4‐expressing or SCP4‐DN‐expressing lentivirus with either FoxO3a‐ or FoxO1‐overexpressing lentivirus into ATDC5 cells. The results showed a significantly lower phosphorylation level of FoxO3a in SCP4‐WT cells compared to SCP4‐DN cells (Figure 4F,G and S2F). Meanwhile, the phosphorylation level of FoxO1 was comparable between SCP4‐WT and SCP4‐DN transfected groups (Figure S2G,H). These results suggest that the dephosphorylation of FoxO3a by SCP4 is essential for the apoptosis of growth plate chondrocytes associated with endochondral osteogenesis during embryonic cartilage development (Figure 5).

## 4 DISCUSSION

In the present study, we demonstrated that the deletion of SCP4, specifically within embryonic chondrocytes, elevated the phosphorylation level of FoxO3a, promoting apoptosis in chondrocytes at different stages and eventually resulting in abnormal endochondral ossification and chondrodysplasia. These findings have identified a novel function of SCP4 in maintaining the homeostasis of chondrocyte differentiation, particularly in the proliferation, hypertrophy, mineralisation and apoptosis of chondrocytes during embryonic cartilage development. This regulatory mechanism is partially dependent on SCP4's dephosphorylation activity on FoxO3a, capable of governing the normal development of cartilage through the chondrocyte apoptosis pathway Further mechanistical analysis using in vivo and in vitro experiments indicated that SCP4 deficiency promotes chondrocyte apoptosis, thus impeding cartilage development. Moreover, models featuring chondrocyte‐specific *SCP4* knockout and *SCP4* global knockout provide additional corroboration of SCP4's indispensability in cartilage development.

Cartilage endochondral ossification commences through mesenchymal cell coalescence and differentiation into cartilage.^28^ With the embryonic growth plate, three layers of chondrocytes, RZ, PZ and HZ, contribute to the longitudinal growth of long bones.^29^ Notably, the enlargement of hypertrophic chondrocytes predominantly governs the growth rate of the inner cartilaginous layer of bone.^5^, ^30^ Cartilage then undergoes a programme involving cell proliferation, maturation, hypertrophic differentiation, calcification, apoptosis and eventual replacement by bone tissue.^31^ Our findings from whole‐mount skeleton staining and histopathological staining underscore that *SCP4* deficiency delays skeletal development in mice, especially in chondrocyte‐specific *SCP4*‐deficient mice. Embryonic chondrocytes across all three regions of *SCP4* ^*Col2ER*^ mice showed varied length shortening as well as reduced cell number and size compared to littermate controls. Moreover, the skull, which is predominantly intramembranous, also has developmental abnormalities. It is reported that Col2^+^ cells include several osteoblasts and suture mesenchymal cells in calvaria,^32^, ^33^ whether SCP4 has a similar function in these cells is unknown. Subsequently, RNA‐sequencing analysis revealed that the specific knockout of SCP4 in chondrocytes not only severely impaired the biological processes of chondrocyte differentiation, cartilage development, ossification and skeletal system development, but also enhanced the apoptosis signalling pathway, a finding was experimentally validated in *SCP4* ^*Col2ER*^ mice and primary chondrocytes via elevated expression of Caspase proteins.

FoxO1 and FoxO3a, as ubiquitously expressed transcriptional factors, are implicated in various biological processes, including cellular differentiation, apoptosis, stem cell homeostasis and metabolism.^34^, ^35^ Specifically, FoxO1 and FoxO3a exert chondroprotective effects crucial for maintaining cartilage homeostasis, whereas dysfunctional FoxO1 and FoxO3a might contribute to chondrodysplasia.^36^, ^37^, ^38^ Moreover, the regulation of FoxO1 and FoxO3a activity hinges on their phosphorylation status, where the dephosphorylated form of FoxOs translocates to the nucleus for transcriptional activation.^39^ Our previous studies have revealed that both FoxO1 and FoxO3a are substrates of phosphatase SCP4, wherein SCP4 can dephosphorylate pFoxO1 and pFoxO3a to promote their nuclear retention, consequently enhancing the glucogenesis‐related gene expression.^40^ Intriguingly, herein, our results revealed a significant increase solely in pFoxO3a level within growth plate chondrocytes in *SCP4* ^*Col2ER*^ mice holding no marked alteration in pFoxO1, suggesting the functional specificity of SCP4, FoxO1 and FoxO3a across diverse physiological processes.

FoxO3a has both proapoptotic and anti‐apoptotic effects in different cellular contexts, depending on whether these cells are unfavourable or favourable to the survival of the organism.^27^, ^41^, ^42^, ^43^ Extensive studies have demonstrated FoxO3a's protective role in chondrocytes by inhibiting the expression of pro‐apoptosis‐related proteins, thus resisting articular cartilage degeneration.^44^, ^45^, ^46^ Consistent with these findings, our results corroborate FoxO3a's regulatory role in counteracting chondrocyte apoptosis in the growth plate during embryonic development. The increased expression of pFoxO3a caused by SCP4 gene deletion in chondrocytes induces FoxO3a dysfunction, which significantly augments chondrocyte apoptosis, thereby affecting the endochondral ossification process and ensuing reduction in bone size. Nevertheless, although this study elucidates the anti‐apoptotic effect of FoxO3a on chondrocytes and identifies SCP4 as the regulator of FoxO3a in chondrocyte development by in vivo and in vitro experiments, whether SCP4 acts on other proteins through this process needs further investigation. Notably, SCP4 plays a key role in gluconeogenesis and chronic kidney disease‐associated muscle wasting and pancreatic cancer.^18^, ^47^, ^48^ Our study notably uncovered SCP4's hitherto unreported function related to bone, providing its potential as a therapeutic target for cartilage‐related diseases.

In conclusion, our results underscore SCP4's pivotal protective role in endochondral ossification and skeletal development, thus allowing normal skeletal development during embryogenesis. The underlying regulatory mechanism might be related to the dephosphorylation activity of the phosphatase SCP4 on FoxO3a, maintaining the transcriptional activity of FoxO3a, and thus regulating chondrocyte apoptosis. Hence, this study uncovered a novel function of SCP4 in regulating cartilage development and endochondral osteogenesis.

## AUTHOR CONTRIBUTIONS

Hongting Jin, Xia Lin, Jianying Feng and Hongfeng Ruan conceived and designed the project. Pinger Wang and Kaiao Zou carried out most of the experiments. Jin Cao and Zhengmao Zhang contributed to data collection. Pinger Wang, Kaiao Zou and Hongting Jin performed the data analysis. Pinger Wang, Jiali Chen and Jianbo Xu performed most of the bioinformatics analysis. Wenhua Yuan and Zhengmao Zhang are responsible for breeding transgenic mice. Pinger Wang and Kaiao Zou wrote and edited the manuscript. Hongting Jin, Xia Lin, Jianying Feng, Di Chen and Hongfeng Ruan contributed to the manuscript revision.

## FUNDING INFORMATION

This study was supported by the National Natural Science Foundation of China (82274550), the Natural Science Foundation of Zhejiang Province (LY22H270005)，the Traditional Chinese Medical Administration of Zhejiang Province (2024ZL052) and the Natural Science Project of Zhejiang Chinese Medical University (2022JKZKTS32).

## CONFLICT OF INTEREST STATEMENT

The authors declare that they have no conflict of interest.

## Supporting information


**Supplemental Figure S1.** A: Length of primary ossification centres in the tibia of SCP4^Col2ER^ and Cre‐negative mice (red line segments). Scale bar: 0.05 mm.
B: Length of mouse tibial growth plates (RZ, PZ and HZ). Scale bar: 0.05 mm.

**Supplemental Figure S2.** A: Heatmap of differential gene expression between Ad‐GFP and Ad‐Cre transfected *SCP4* ^*f/f*^ primary chondrocytes.
B: Volcano plots of differentially expressed genes in *SCP4* ^*f/f*^ primary chondrocytes transfected with Ad‐Cre or Ad‐GFP. The green and red dots represent the down‐ and up‐regulated genes, respectively.
C: Ad‐GFP and Ad‐Cre group differential gene bias.
D: Representative immunofluorescence staining of pFoxO1 (green) in the growth plate of E16.5. DAPI (blue) was used for nuclear staining.
E: Quantification of the data from immunofluorescence staining.
F: Quantification of the data from the Western blot of FoxO3a.
G: SCP4 does not dephosphorylate pFoxO1. ATDC5 cells were co‐transfected with Lv‐FoxO1 and Ad‐SCP4 (SCP4‐WT) or Lv‐SCP4‐DN. The levels of pFoxO1, total FoxO1 and SCP4 were determined by Western blot.
H: Quantification of the data from Western blot.

## Data Availability

Data are available from corresponding author upon reasonable request.
