# Supplementary figures and images for: Protein phosphatase SCP4 regulates cartilage development and endochondral osteogenesis via FoxO3a dephosphorylation
Source: Cell Prolif. 2024 Jun 17;57(9):e13691. doi: 10.1111/cpr.13691 (PMC11503251; doi:10.1111/cpr.13691)

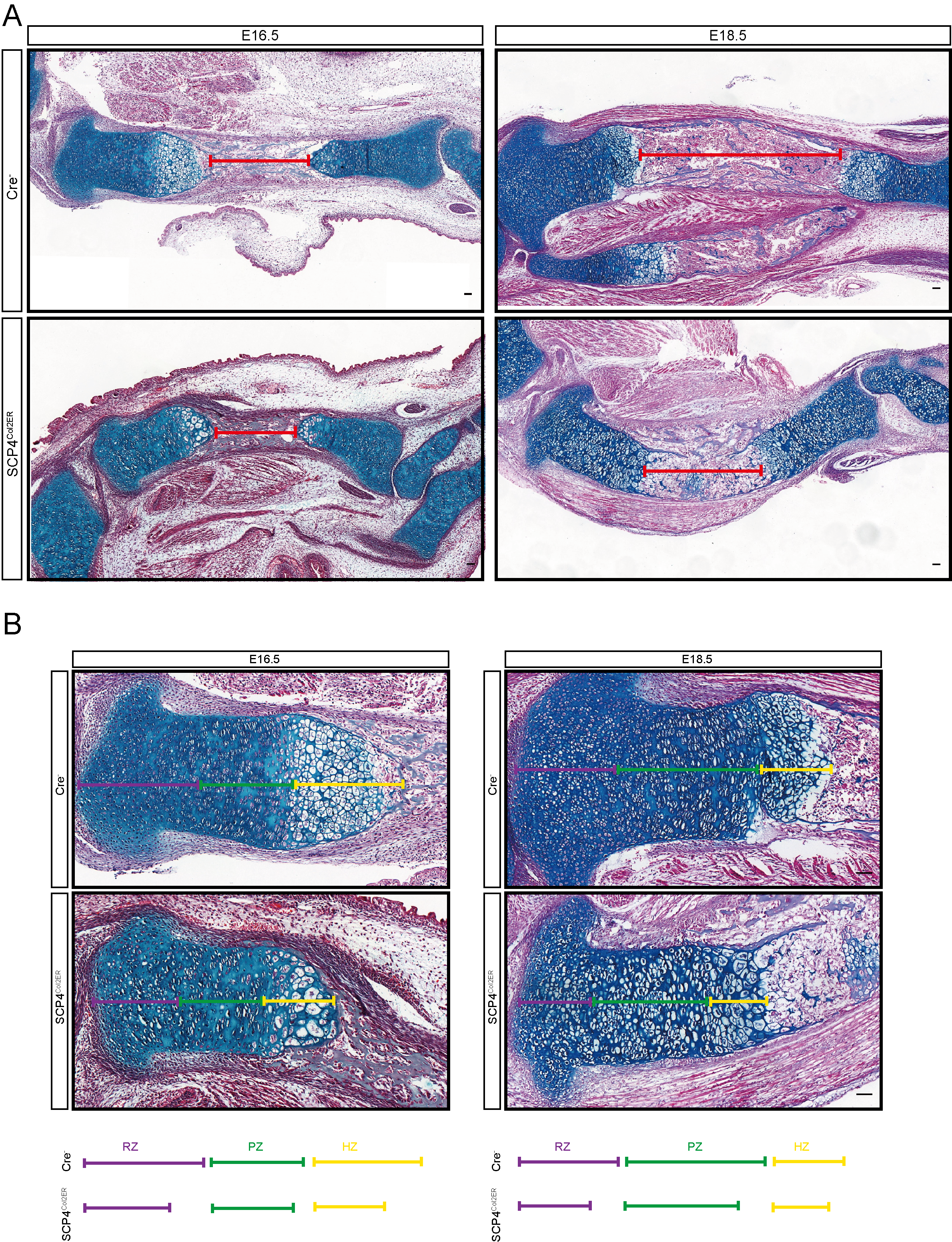

Supplement: Supplementary file 1 — Supplemental Figure S1. A: Length of primary ossification centres in the tibia of SCP4Col2ER and Cre‐negative mice (red line segments). Scale bar: 0.05 mm. B: Length of mouse tibial growth plates (RZ, PZ and HZ). Scale bar: 0.05 mm. [file CPR-57-e13691-s002.tif]

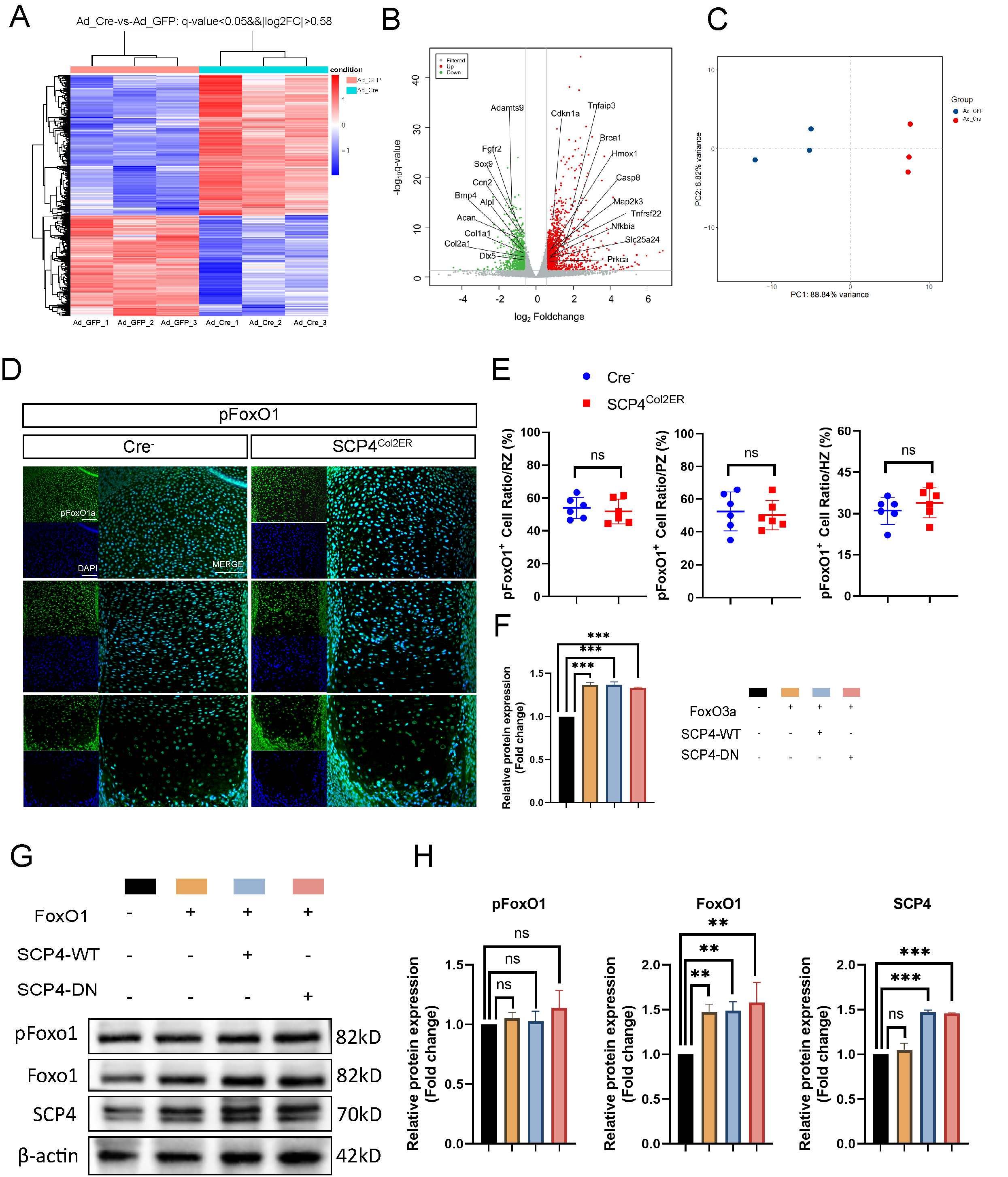

Supplement: Supplementary file 2 — Supplemental Figure S2. A: Heatmap of differential gene expression between Ad‐GFP and Ad‐Cre transfected SCP4 f/f primary chondrocytes. B: Volcano plots of differentially expressed genes in SCP4 f/f primary chondrocytes transfected with Ad‐Cre or Ad‐GFP. The green and red dots represent the down‐ and up‐regulated genes, respectively. C: Ad‐GFP and Ad‐Cre group differential gene bias. D: Representative immunofluorescence staining of pFoxO1 (green) in the growth plate of E16.5. DAPI (blue) was used for nuclear staining. E: Quantification of the data from immunofluorescence staining. F: Quantification of the data from the Western blot of FoxO3a. G: SCP4 does not dephosphorylate pFoxO1. ATDC5 cells were co‐transfected with Lv‐FoxO1 and Ad‐SCP4 (SCP4‐WT) or Lv‐SCP4‐DN. The levels of pFoxO1, total FoxO1 and SCP4 were determined by Western blot. H: Quantification of the data from Western blot. [file CPR-57-e13691-s001.tif]
